# Challenges of Robot‐Assisted Surgery in Reconstructive Microsurgery: A Scoping Review

**DOI:** 10.1002/micr.70339

**Published:** 2026-11-10

**Authors:** Lewei Cai, Ishith Seth, Sophie Ricketts, Eldon Mah

**Affiliations:** ^1^ Melbourne Medical School The University of Melbourne Melbourne Victoria Australia; ^2^ Plastics, Reconstructive and Hand Surgery St Vincent's Hospital Melbourne Victoria Australia

**Keywords:** learning curve, microsurgery, plastic surgery, review literature, robotics

## Abstract

**Introduction:**

Robotic platforms have been popularized in reconstructive microsurgery for their ability to enhance precision and dexterity, filter tremor, and reduce surgical fatigue. Nonetheless, implantation challenges compared with conventional methods remain poorly synthesized. This scoping review identifies common barriers to robotic platforms in reconstructive microsurgery.

**Method:**

A literature search was conducted in PubMed (Medline), Embase, and the Cochrane Library for English‐language publications between January 1, 2016 and September 30, 2026 reporting clinical challenges in robot‐assisted surgical reconstruction. Publications included were primary research. Reviews and preclinical studies were excluded. A total of 726 articles were screened, and 30 met the inclusion criteria.

**Results:**

Thirty peer‐reviewed publications were reviewed, predominantly from European and American institutions utilizing the Da Vinci Surgical System, Medical Microinstrument's Symani Surgical System, or MicroSure's MUSA surgical robots. Most consistently reported challenges were prolonged operative and anastomotic times in 83% of studies, increased operative costs in 57% of studies, learning and adaptation to robot‐assisted microsurgery, as well as a lack of haptic feedback and complex operating room set‐up. Less commonly reported barriers include instrument reliability, physiological motion impairing operative control, large robotic footprint, sustainability of single‐use consumables, limited operative field, and magnetic interference. Inconsistent reporting on the learning curve of robot‐assisted surgery was noted. Overall, the evidence base is limited by conflicts of interest with robotic manufacturers present within 37% of reviewed studies, small sample sizes, methodological heterogeneity, and paucity of long‐term patient outcomes. These limitations may underestimate the challenges surgeons face in adopting robotic platforms in reconstructive microsurgery.

**Conclusion:**

Robot‐assisted reconstructive microsurgery is a promising but maturing field. Future research should include manual microsurgical control groups and report long‐term clinical outcomes to strengthen the evidence base for robot‐assisted reconstructive microsurgery. Randomized controlled trials with manual control groups will help guide the standardization of robot‐assisted microsurgery training curricula.

## 1 Introduction

Microsurgery is a specialized surgical technique that uses an operating microscope to enable precise visualization of fine anatomical structures. Within reconstructive surgery, microsurgery underpins the surgical management of complex tissue defects.

With rapid technological development, robotic platforms have challenged traditional barriers of manual microsurgery. Since the first described case of robot‐assisted neurosurgical biopsy in 1985, robot‐assisted surgery has been welcomed across multiple surgical specialties through enhancing precision and dexterity, filtering tremor and reducing surgical fatigue (Kwoh et al. 1988; Reddy et al. 2023). The introduction of the Da Vinci Surgical System in 1998 has predominated current robot‐assisted surgical practice, with widespread adoption across multiple surgical disciplines (Morrell et al. 2021). In microsurgery, the need for refined movement led to the development of two robotic platforms: MicroSure's MUSA in 2009 and the Symani Surgical System in 2015, both now used predominantly in Europe and North America (Barbon et al. 2022; van Mulken et al. 2020; Struebing, Boecker, et al. 2024). The Symani Surgical System features a portable cart with flexible robotic arms, wrist‐mounted microsurgical instruments, and a separate surgeon console that allows remote operation away from the surgical field. In contrast, the MUSA surgical system is not designed for telesurgery. It mounts directly onto a surgical scaffold, where conventional microsurgical instruments attach to adapters on the robotic platform. The surgeon controls the console beside the operating table.

Robot‐assisted microsurgery now includes microvascular anastomosis, lymphatic reconstruction, and nerve coaptation. These techniques have become indispensable across various clinical contexts (Figure 1).

**FIGURE 1:**
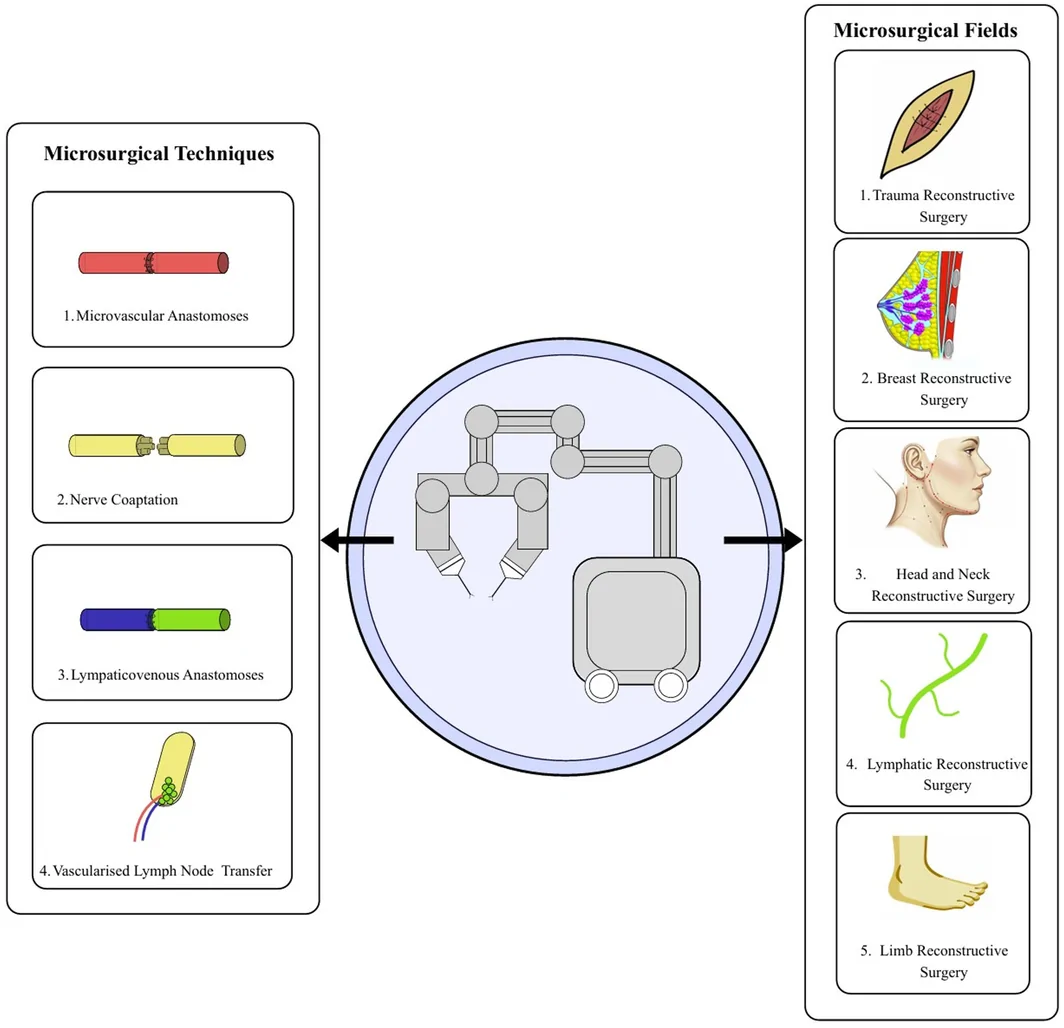
Representation of the current common techniques and microsurgical fields of robot‐assisted reconstructive microsurgery.

Despite the potential advantages of microsurgery, multiple systematic reviews have reported similar patient outcomes between conventional and robot‐assisted microsurgery. Specifically, anastomotic patency and complication rates are similar (Seth et al. 2025; Awad et al. 2024; Brown et al. 2025).

The literature on limitations and challenges of robot‐assisted reconstructive microsurgery also remains limited. Existing systematic reviews conducted in this field have yielded several key barriers, including increased operating time, high cost, lack of haptic feedback, “stickiness” of instruments, poor visibility, large footprint of the robot and a large surgeon learning curve (Seth et al. 2025; Brown et al. 2025). However, these have been identified as secondary findings within broader analysis of the robotic applications rather than the primary focus. With the rapid publication of this novel technology in plastic and reconstructive surgery, this scoping review aims to bridge this gap by examining the existing literature. This study aims to synthesize findings on the challenges of robot‐assisted surgery in reconstructive microsurgery and identify knowledge gaps to guide future research priorities.

## 2 Materials and Methods

This scoping review was structured with the Population/Participants, Concept/Context (PCC) Question: For surgeons performing robot‐assisted microsurgery, what are the challenges and barriers with utilizing robot‐assisted platforms in reconstructive microsurgical procedures?

The review was conducted in accordance with the Preferred Reporting Items for Systematic Reviews and Meta‐Analyses Extension for Scoping Reviews (PRISMA‐ScR) guidelines. A comprehensive literature search was undertaken to identify studies reporting the challenges, limitations, and barriers associated with robot‐assisted reconstructive microsurgery. Electronic searches were performed in PubMed (MEDLINE), Embase (Ovid), and the Cochrane Library. The final search was conducted on September 30, 2026. The search strategy combined controlled vocabulary and free‐text terms relating to robot‐assisted surgery, reconstructive microsurgery, and microsurgical anastomosis. A generic search strategy is presented in Table 1 (a detailed replicable search strategy for each database is provided in Table S1).

**TABLE 1:** Outline of search strategy with key search terms and method of literature selection.

Aspect	Details
Date of search	30/09/2026
Databases searched	PubMed (MEDLINE), Cochrane, Ovid (Embase)
Key search terms	(limitation* or drawback* or learn* or barrier* or experience*) AND (robot* or Symani* or MUSA or MicroSure or DaVinci) AND (microsurg* or microvascular* or “free flap” or ((vascul* or lymph*) and anastom*) AND (repair* or revascular* or reconstruct*))
Literature selection	The first and second authors independently conducted the selection, and they resolved any disagreements through discussion. This search produced 726 results. After applying inclusion/exclusion criteria and removing duplicates, we reviewed 30 publications.

The search was restricted to studies published between January 1, 2016 and September 30, 2026, representing the most recent 10 years of literature. This timeframe captured evidence on contemporary robotic platforms and technological developments while excluding earlier studies evaluating obsolete systems with limited relevance to current clinical practice. After removing duplicate records, two reviewers independently screened titles and abstracts against the predefined eligibility criteria. Full‐text articles considered potentially relevant were subsequently assessed independently by both reviewers. Reviewers resolved any disagreements about study eligibility through discussion and consensus.

The PRISMA‐ScR flow diagram (Figure 2) presents the study selection process. Eligibility criteria were defined a priori. Only studies published in English or translated to English from January 1, 2016 to September 30, 2026 were included. Clinical studies involving human participants were eligible, whereas animal and preclinical studies were excluded because their findings may not accurately reflect the technical and clinical challenges encountered during human reconstructive microsurgery. Studies were required to evaluate the application of robotic systems in reconstructive microsurgery. We excluded studies from other surgical specialties because differences in operative techniques and workflows may limit the transferability of findings. Eligible studies were required to report at least one outcome relating to the challenges, limitations, barriers, or technical difficulties experienced by the operating team during robot‐assisted reconstructive microsurgery. Original primary research studies of any design were eligible for inclusion, while review articles, editorials, commentaries, conference abstracts, and correspondence were excluded. The first and second authors jointly evaluated the risk of bias for the analyzed articles. All case series were evaluated using the JBI Critical Appraisal Checklist for Case Series (Table S2); all randomized controlled trials were evaluated with the RoB 2 tool (Table S3); cross‐sectional studies were evaluated with the JBI cross‐sectional studies tool (Table S4); while cohort and case–control studies were evaluated with the Newcastle‐Ottawa scale (Table S5). Any discrepancies were resolved through discussion and consensus.

**FIGURE 2:**
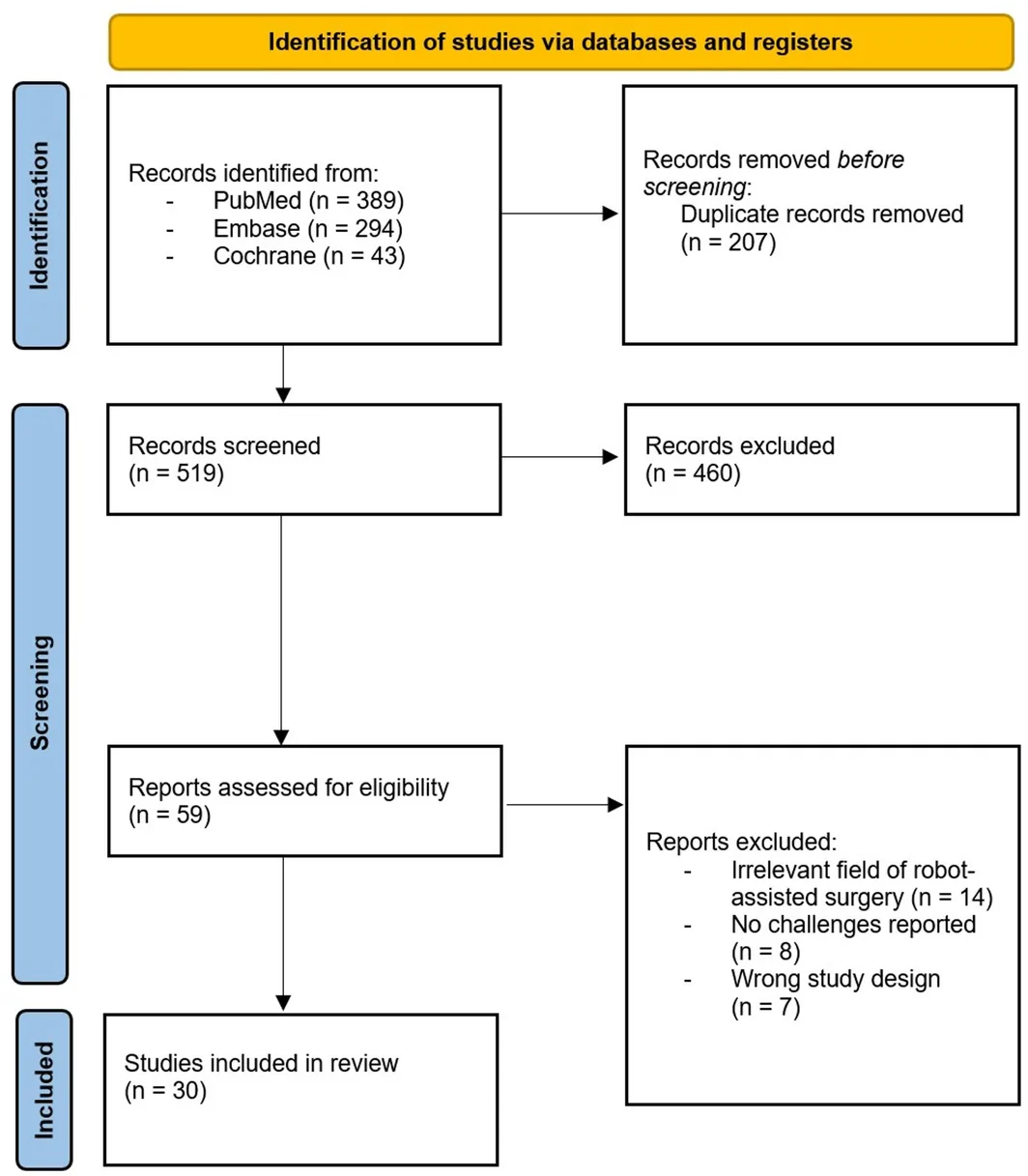
PRISMA diagram of study selection.

The first and second authors independently extracted data using a standardized data extraction form in covidence, capturing study design, sample size, field of robot‐assisted microsurgery, robotic platform used, key challenges reported by each study, and any noted conflicts of interest. Reviewers resolved discrepancies through discussion and consensus.

## 3 Results

### 3.1 Characteristics of Selected Studies

Thirty peer‐reviewed publications were synthesized (*n* = 30). Study designs included retrospective and prospective case series, randomized pilot trials, retrospective cohort studies, a cross‐sectional survey, and a case–control study (Figure 3). Most articles described the application of robot‐assisted microsurgery in free flaps (*n* = 21) and lymphatic surgery (*n* = 17). Seven studies (*n* = 7) also reported using a surgical robot in nerve repair. Many studies reported findings across multiple applications (Figure 4).

**FIGURE 3:**
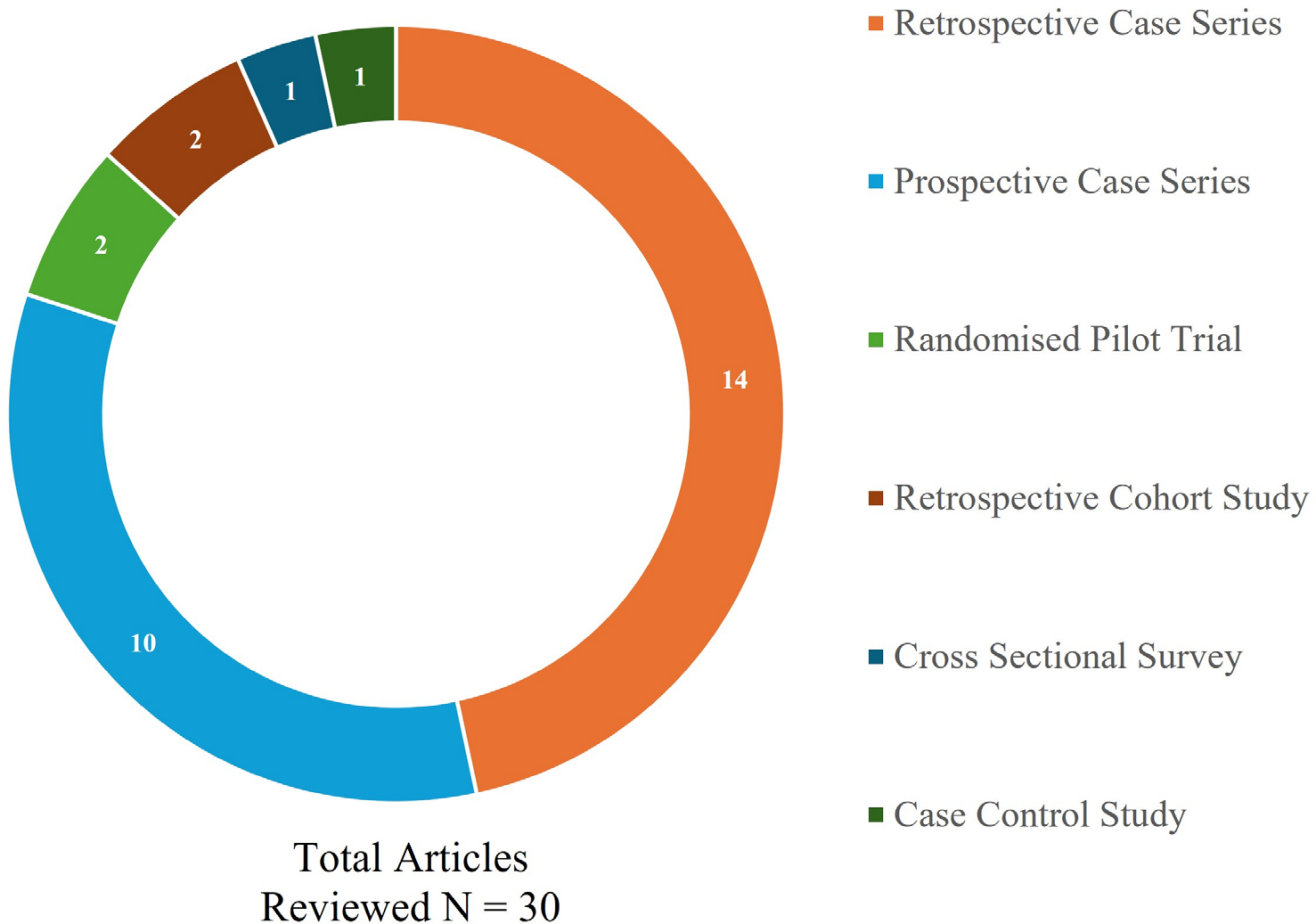
Pie graph representation of the study designs of articles reviewed.

**FIGURE 4:**
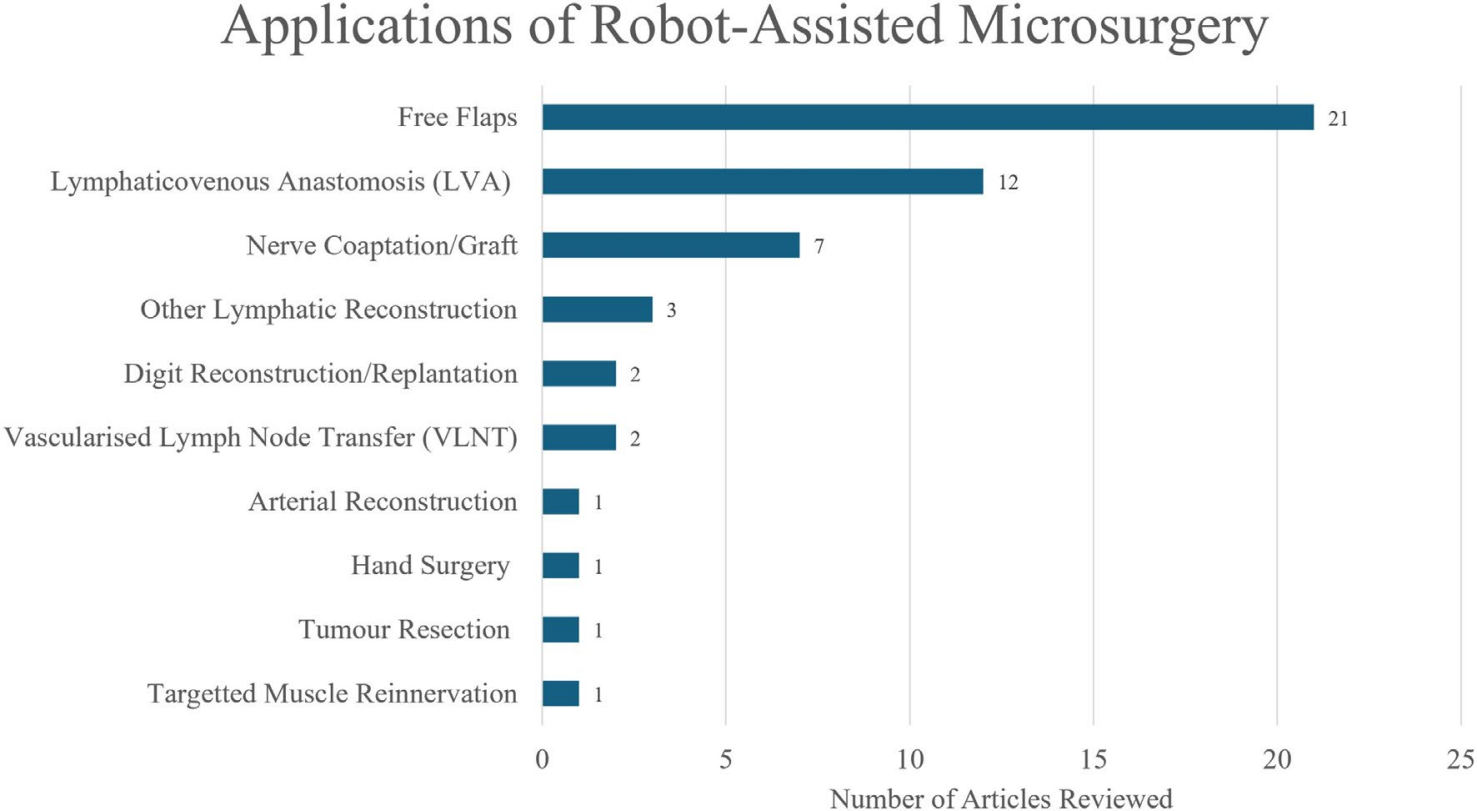
Bar graph representation of the applications of robot‐assisted microsurgery described by the articles reviewed. Some reviewed articles discussed more than one application of robot‐assisted reconstructive microsurgery.

### 3.2 Geographic and Commercial Distribution

A significant theme identified was the geographic and commercial concentration of the current evidence. All publications (*n* = 30) originated from authors operating in European or American institutions utilizing one of three platforms: the Da Vinci Surgical System, the Symani Surgical System, or MicroSure's MUSA surgical system.

### 3.3 Reported Challenges

Overall, the most common challenges (Table 2) include workflow and economic challenges, including increases in average anastomotic and operational times and related increases in operational costs. Technical challenges such as the lack of haptic feedback, limited instrument reliability, difficulties with robotic training, ergonomics, and operating room set‐up were also widely reported. Table 3 provides a more detailed analysis of all challenges reported by each reviewed article. Table 4 provides a summary of the average anastomotic times and patency reported by each article.

**TABLE 2:** Frequency table of commonly reported challenges in robot‐assisted reconstructive microsurgery.

Challenge reported	Reported frequency	Percentage (%) of reviewed articles
Prolonged anastomotic/operational time as compared with manual surgery	25	83%
High running/operational costs	17	57%
Challenges with learning and training	14	47%
Challenges with operating room set‐up	10	33%
Lack of haptic feedback	10	33%
Challenges with instruments	7	23%
Challenges with chest movement (physiological cardiorespiratory movements in surgery in the thoracic field)	4	13%
Ergonomic discomfort	4	13%
Sustainability concerns	4	13%
Limited range of motion of robot and operative field	3	10%
Magnetic interference from surrounding environment	3	10%

*Note:* Only challenges reported in more than two articles are included.

**TABLE 3:** Summary of key challenges identified from included studies.

Author and date of publication	Application of microsurgery	Number of patients in study	Robotic platform	Key robot‐assisted surgical challenges identified	Conflicts of interest with robotics company
Lai et al. (2019)	Free flap	*N* = 15	Da Vinci Surgical System	Lack of haptic feedback; prolonged anastomotic and set‐up time; increased operating costs	No
van Mulken et al. (2020)	LVA	*N* = 12 (manual control) *N* = 8 (robot‐assisted)	MUSA Surgical System	Prolonged operational time; prolonged anastomotic time; poorer microsurgical skills score of anastomoses rated by blinded independent microsurgeons	Yes (with MicroSure)
Barbon et al. (2022)	Free flap, lymphatic, nerve repair	*N* = 22	Symani Surgical System	Prolonged anastomotic time; increased number of stitches; lack of haptic feedback; difficulty in operating room set‐up; magnetic interference with the operating table	Yes (with MMI)
Lindenblatt et al. (2022)	Free flap, VLNT, LVA, tumor resection	*N* = 5	Symani Surgical System	Prolonged operative duration; lack of haptic feedback; necessity of robotic‐specific training; high running costs; sustainability concerns with single‐use instruments	Yes (with MMI)
van Mulken et al. (2022)	LVA	*N* = 20	MUSA Surgical System	Lower LVA patency rates in robotic cases at 1 year; prolonged anastomotic and operational time; increased running costs; presence of a learning curve	Yes (with MicroSure)
Wittesaele and Vandevoort (2022)	Free flap	*N* = 10	Da Vinci Surgical System	Prolonged operational and set‐up time; difficulty with operating room set‐up; steep learning curve; increased operating costs	No
Daar et al. (2022)	Free flap	*N* = 4	Da Vinci Surgical System	Increased operating costs; prolonged operational time	No
Beier et al. (2023)	Free flap	*N* = 23	Symani Surgical System	Lack of haptic feedback; prolonged anastomotic time	No
Shin et al. (2023)	Free flap	*N* = 22	Da Vinci Surgical System	Prolonged operational time	No
Weinzierl et al. (2023)	VLNT, LVA	*N* = 8	Symani Surgical System	Prolonged operational time; increased operational and running costs	Yes (with MMI)
Kueckelhaus (2024)	Free flap	*N* = 4	Symani Surgical System	Slow learning curve; prolonged operational time; necessity of robotic‐specific training	Yes (with MMI)
Struebing, Boecker, et al. (2024)	Free flap, lymphatic, nerve repair	*N* = 100	Symani Surgical System	Difficulty in needle passage through atherosclerotic vessel; lack of haptic feedback; prolonged anastomotic time; some device malfunctions	No
Struebing, Bigdeli, Weigel, et al. (2024)	Free flap, LVA, nerve repair	*N* = 50	Symani Surgical System	Increased operating costs attributed to increased operating times; lack of haptic feedback	No
Wessel et al. (2024)	Free flap	*N* = 28	Symani Surgical System	Prolonged anastomotic, ischemic and total operational time; magnetic interference with electrical implants; difficulty with operating room set‐up; issues with chest movement	Yes (with MMI)
Gorji et al. (2024)	Free flap	*N* = 23	Symani Surgical System	Steep learning curve; difficulties with chest movement; collisions between optical and robotic systems; increased running costs	No
Dastagir et al. (2024)	Digit reconstruction and replantation	*N* = 21 (10 robot‐assisted, 11 manual)	Symani Surgical System	Presence of an initial learning curve; crowding of the operating room; increased operational costs	No
Struebing, Bigdeli, Boecker, et al. (2024)	Free flap	*N* = 16	Symani Surgical System	Increased operational costs; prolonged anastomotic times; steep learning curve; single‐use instruments and impacts on sustainability	No
Aman et al. (2024)	Peripheral nerve surgery	*N* = 19	Symani Surgical System	Difficulty with operational room set‐up; limited surgical equipment; stickiness of instruments; single‐use instruments and impacts on sustainability	No
Moreira et al. (2025)	Free flap	*N* = 47 (23 robot‐assisted, 24 manual)	Da Vinci Surgical System	Prolonged operational time; increased operational costs	No
von Reibnitz et al. (2024)	Lymphatic reconstruction (LVA; lympho‐lymphatic anastomoses; lymphatic tissue transfer)	*N* = 67	Symani Surgical System	Prolonged operational and anastomotic time; necessity of robotic‐specific training; difficulty with operating room set‐up; increased operational costs	Yes (with MMI)
Struebing, Kneser, et al. (2025)	Free flap, nerve and vessel reconstruction; LVA	*N* = 87	Symani Surgical System	Ergonomic discomfort; prolonged operational time	No
Watson et al. (2025)	Free flap	*N* = 6	Symani Surgical System	High operational and training costs; lack of haptic feedback; prolonged anastomotic time; stickiness of instruments; magnetic interference with operating table	Yes (with MMI)
Piperno et al. (2025)	Hand surgery	*N* = 8	Symani Surgical System	Prolonged operating room set‐up time; necessity of robotic‐specific training	No
Chen et al. (2025)	Lymph node to vein anastomosis	*N* = 22	Symani Surgical System	Difficulty adapting to scaling and limited operative field; flat learning curve	Yes (with MMI)
Struebing, Gazyakan, et al. (2025)	Free flap, LVA, nerve repair, targeted muscle reinnervation	*N* = 85	Symani Surgical System	Difficulty with operational room set‐up; stickiness of instruments; lack of haptic feedback; high operational costs; prolonged anastomotic time; difficulties with chest movement; difficulty in needle passage through atherosclerotic vessels	No
Mayrhofer‐Schmid et al. (2025)	Not applicable	Not applicable	Symani Surgical System, MUSA Surgical System	Lack of haptic feedback; prolonged operational time; slow learning curve; poor visibility and range of motion	No
Spille et al. (2025)	Free flap	*N* = 93	Symani Surgical System	Steep learning curve; prolonged anastomotic time; prolonged operating room set‐up time	No
Sørensen et al. (2025)	Free flap, LVA	*N* = 12	Symani Surgical System	Ergonomic discomfort due to constrained operating positions with the microscope and robot; difficulties with chest movement; concerns about reducing manual experience for training reconstructive surgeons	No
Bettens et al. (2026)	LVA, free flap, digital nerve repair, thumb replantation	*N* = 7	MUSA Surgical System	Lack of haptic feedback; prolonged operational time; increased operational costs; slow learning curve	Yes (with MicroSure)
Struebing et al. (2026)	Free flap	*N* = 18	Symani Surgical System	Prolonged anastomotic time; increased operational costs; single‐use instruments and impacts on sustainability	No

*Note:* Any conflicts of interest with robotic manufacturers are also listed.

**TABLE 4:** Reported average robot‐assisted anastomotic times as well as intraoperative patency rates of robot‐assisted anastomosis.

First author and year	Reported average robotic‐assisted anastomotic times	Overall intraoperative robotic anastomosis patency
Lai et al. (2019)	38.4 ± 10.4 min (mixed anastomosis)	100%
van Mulken et al. (2020)	25 ± 6 min (LVA)	100%
Barbon et al. (2022)	25.3 ± 12.3 min (mixed anastomosis)	97.5%
Lindenblatt et al. (2022)	Not reported	100%
Beier et al. (2023)	Ranges from 50 ± 8 min (end to side arterial anastomosis) to 69 ± 56 min (end to end arterial anastomosis)	Not reported
Weinzierl et al. (2023)	22.6 ± 26.2 min (VLNT and LVA)	100%
Kueckelhaus (2024)	25 ± 5 min (arterial anastomosis)	100%
Struebing, Boecker, et al. (2024)	Ranges from 28.1 ± 11.4 min (venous anastomosis) to 38.3 ± 14.5 min (LVA)	Not reported
Struebing, Bigdeli, Weigel, et al. (2024)	Ranges from 23.8 ± 9.2 min (venous anastomosis) to 37.4 ± 13.9 min (end to side arterial anastomosis)	Not reported
Wessel et al. (2024)	29 ± 12 min (arterial anastomosis)	100%
Gorji et al. (2024)	36.7 ± 10.9 min (arterial anastomosis)	95.7%
Dastagir et al. (2024)	Ranges from 11.5 ± 1.3 min (venous anastomosis) to 17.3 ± 1.9 min (arterial anastomosis)	100%
Struebing, Bigdeli, Boecker, et al. (2024)	Ranges from 20 ± 6 min (venous anastomosis) to 33 ± 13 min (arterial end to side anastomosis)	Not reported
Watson et al. (2025)	32.5 ± 13.5 min (arterial anastomosis)	100%
Chen et al. (2025)	Between 18 and 37 min (lymphatic anastomosis)	100%
Spille et al. (2025)	Around 20 to 30 min (mixed anastomosis)	Not reported
Sørensen et al. (2025)	38.5 ± 19.5 min (mixed anastomosis)	Not reported
Struebing et al. (2026)	25.7 ± 4.2 min (end to end arterial anastomosis) to 36 min (end to side arterial anastomosis)	Not reported

*Note:* Only articles with published anastomotic times were included.

### 3.4 Risk of Bias

Notably, 11 of the 30 studies (37%) disclosed a conflict of interest with the relevant robotic manufacturer, a large proportion that requires consideration when interpreting reported challenges.

## 4 Discussion

Overall, the literature identifies robot‐assisted microsurgery as a promising tool for developing reconstructive microsurgery. However, several challenges may limit widespread adoption.

### 4.1 Haptic Feedback

Many authors report that dedicated robotic microsurgical platforms currently lack haptic feedback. Lindenblatt et al. first identified this issue in an early case series in 2022, conducted on the Symani system for lymphatic reconstruction. Nevertheless, the primary author suggested surgeons could quickly develop a “see‐feel” technique to infer mechanical forces applied to tissue (Lindenblatt et al. 2022). The impact of this should not be understated. In a similar study, vascular intimal damage was attributed to a lack of haptic feedback, demonstrating the inherent risks of robot‐assisted surgery despite the ability to compensate visually (Barbon et al. 2022). Similar challenges have been reported in case series of free tissue transfer, where surgeons have experienced difficulty with knot tying in the absence of tactile feedback, arguing that visual feedback alone may be less reliable (Beier et al. 2023). In a study by Watson et al. (2025) this disadvantage is cited as contributing to longer anastomotic times. Severely atherosclerotic and scarred vessels also complicated suturing without haptic feedback and sufficient force control (Struebing, Boecker, et al. 2024; Struebing, Bigdeli, Weigel, et al. 2024; Struebing, Gazyakan, et al. 2025). The repeated reports of a lack of haptic feedback from multiple authors suggest this may be a common challenge for beginners in robot‐assisted surgery. This is supported by a 2025 cross‐sectional survey of robotic microsurgeons, in which 85.7% noted a lack of haptic feedback as a disadvantage of robot‐assisted surgery (Mayrhofer‐Schmid et al. 2025). Overall, despite the difficulty of objectively comparing outcomes with reduced haptic feedback in robot‐assisted microsurgery to conventional microsurgery, this appears to be an important, widely reported challenge across all reviewed robotic platforms and may affect operative safety and surgeon confidence (Lai et al. 2019; Bettens et al. 2026).

### 4.2 Instrument Reliability and Physiological Motion

A further challenge involves instrument reliability. Barbon et al. (2022) reported instruments becoming “sticky” with prolonged use when analyzing the early learning curve of the Symani system. This was later reinforced through further case series (Aman et al. 2024; Watson et al. 2025; Struebing, Gazyakan, et al. 2025). Aman et al. (2024) also cite that the limited available range of microsurgical equipment poses difficulties in nerve surgery. Consequently, these findings suggest that instrument reliability can compound inefficiencies during early use. With the prevalence of free flaps in reconstructive breast surgery, concerns have been raised about the impact of relative movement of the operative surface due to respiration and cardiac motion (Wessel et al. 2024; Gorji et al. 2024; Struebing, Gazyakan, et al. 2025; Sørensen et al. 2025). As such, physiological motion remains an inherent limitation that may reduce the technical reliability of suture placement, as most surgeons performing DIEP surgery report. However, publications on microsurgery in other, less mobile regions did not report similar challenges.

### 4.3 Operating and Anastomotic Times

Almost universally, robot‐assisted reconstructive microsurgery has required longer operative and anastomotic times than manual surgery. This is especially true in the initial learning stage. A randomized pilot trial by van Mulken et al. (2020) on the MUSA system showed that, on average, anastomosis duration for LVAs was significantly longer in robotic cases than in manual cases (25 ± 6 vs. 9 ± 6 min). This study is notable because other publications lacked randomization and direct comparisons between robot‐assisted and manual methods. Prolonged anastomotic and operative times are similarly widely reported in case series across lymphatic and free flap microsurgery but lack the ability to permit a direct, valid comparison with manual methods (Barbon et al. 2022; Struebing, Boecker, et al. 2024; Lai et al. 2019; Lindenblatt et al. 2022; Wittesaele and Vandevoort 2022; Daar et al. 2022; Beier et al. 2023; Shin et al. 2023; Weinzierl et al. 2023; Kueckelhaus 2024; Struebing, Bigdeli, Weigel, et al. 2024; Wessel et al. 2024; Moreira et al. 2025; von Reibnitz et al. 2024; Struebing, Kneser, et al. 2025; Watson et al. 2025; Piperno et al. 2025; Spille et al. 2025; Bettens et al. 2026; Struebing et al. 2026).

### 4.4 Surgical Outcomes

Despite prolonged anastomotic time being a challenge, time can be significantly reduced during the initial learning curve, with Barbon et al. (2022) achieving times comparable to hand‐sewn anastomosis in a single‐center case series after an 8‐month clinical learning period. Objectively, a blinded scoring of anastomosis from van Mulken et al.'s (2020) pilot study also found a statistically significant reduction in anastomosis quality in the robotic group, as perceived by blinded external surgical reviewers, compared with manual methods. Nonetheless, most published literature does not report short‐term patency as an issue with robot‐assisted microsurgical anastomosis. Long‐term patency is less frequently assessed, but in a publication by van Mulken et al. (2022) following their original pilot trial, they reported a lower 1‐year patency rate for robotic LVAs than for manual LVAs (66.6% vs. 81.1%).

### 4.5 Operative Room Footprint and Ergonomics

Another major concern appears to be the large footprint of robotic platforms in the operating environment which requires specialized training of operative staff (Lai et al. 2019; Wittesaele and Vandevoort 2022; von Reibnitz et al. 2024; Piperno et al. 2025). This is especially true in microsurgery, where an optical system is also required. As Gorji et al. described in a 2024 case series on remote (telemetric) robot‐assisted microsurgery, collisions between these two systems were possible, with implications for surgical safety and workflow (Gorji et al. 2024). Similar observations were noted during free flap, LVA, nerve surgery, and replantation surgeries conducted by surgeons in the operating theater, where ergonomic difficulties caused discomfort during prolonged operations (Dastagir et al. 2024; Aman et al. 2024; Struebing, Kneser, et al. 2025; Sørensen et al. 2025). Therefore, these observations suggest challenges in effectively setting up the operating environment in robot‐assisted surgery (Lai et al. 2019).

Furthermore, the Symani Surgical System uses a magnetic field to translate surgeon hand movements into robotic movements, requiring modifications to patient positioning or the operating table to prevent magnetic interference (Barbon et al. 2022; Wessel et al. 2024; Watson et al. 2025).

### 4.6 Cost and Sustainability

Most publications agree that robot‐assisted microsurgery has increased surgical costs, with many citing the direct increase in single‐use operating equipment costs, which can exacerbate healthcare disparities (Lai et al. 2019; Wittesaele and Vandevoort 2022; Daar et al. 2022; Kueckelhaus 2024; Gorji et al. 2024; Dastagir et al. 2024; Struebing, Bigdeli, Boecker, et al. 2024; Moreira et al. 2025; von Reibnitz et al. 2024; Watson et al. 2025; Struebing, Gazyakan, et al. 2025; Bettens et al. 2026; Struebing et al. 2026). However, the heterogeneity of these publications' limits comparison, as equipment prices depend on the surgical procedure. Most publications also lack a control manual microsurgical group, preventing a direct cost comparison to manual methods. Without direct comparison, it is challenging to determine whether increased expenditure reflects the robotic platform itself or confounding factors such as greater case complexity or longer operative time. This is supported by a 2023 case series by Weinzierl et al. (2023), which reports prolonged operative time, surgical staff training, and robotic platform maintenance as additional costs that contribute to the socioeconomic burden on the healthcare system. This systems perspective provides a more holistic view of the cost–benefit analysis of robot‐assisted surgery, mirrored by Struebing, Bigdeli, Boecker, et al. (2024), which attributed the increased cost mostly to increased operating times. However, these publications analyze robot‐assisted reconstructive microsurgery in an early adoption phase, so inefficiencies are expected. As technology matures, robot‐assisted microsurgery may become more cost‐effective. The reviewed studies also focus on European and American healthcare systems; as a result, this limited global representation limits the generalizability of the costs associated with robot‐assisted reconstructive microsurgery.

A less explored but clinically relevant concern is the environmental sustainability of robot‐assisted microsurgery, particularly given the Symani system's dependence on single‐use consumables (Lindenblatt et al. 2022; Struebing, Bigdeli, Boecker, et al. 2024; Aman et al. 2024; Struebing et al. 2026). Whether MMI follows the path other robotic platforms, such as Da Vinci, have taken by introducing reusable equipment remains to be seen, but it would be critical for widespread, sustainable adoption.

### 4.7 Training Standardization

Reporting on the steepness of the initial learning curve for robotic platforms is inconsistent. This appears to be a major gap in the literature, with most studies reflecting the experience of a single highly experienced microsurgeon transitioning to robot‐assisted techniques. As a result, interoperator variability is poorly appreciated, and the generalizability of reported learning trajectories is limited. Some studies report a steep learning curve initially but rapid reductions in operative and anastomotic times, both of which improve with experience. Barbon et al. (2022) found a decrease of average robotic anastomotic times from 23.9 ± 6.8 min (August–December 2021) to 16.3 ± 6.1 min (January–April 2022). Similarly, Lindenblatt et al. (2022) and Bettens et al. (2026) both suggested that long‐standing experience in manual microsurgery benefits adaptation to robotic platforms. Chen et al. (2025) further suggest that despite initial difficulties adapting to operative scaling and the Symani system's small operative field, the initial learning curve in supermicrosurgery quickly flattens. Experience with older robotic platforms such as Da Vinci has also been reported to facilitate quick adaptation to newer surgical systems such as MMI's Symani (Kueckelhaus 2024).

In contrast, Lai et al. (2019) and Spille et al. (2025) emphasized that despite decreases in anastomotic times with experience, additional operating room set‐up time did not significantly decrease, even after repeated use. The reviewed literature also poorly analyses the impact of prior microsurgical experience on the robot‐assisted surgery learning curve, as it includes only experienced senior surgeons, with both Barbon et al. (2022) and Spille et al. (2025) suggesting the need to introduce robotic training for surgeons of varying experience.

Moreover, the standardization of current robotic training programs is also reported as a concern (Struebing, Boecker, et al. 2024; Mayrhofer‐Schmid et al. 2025), with training offered differing vastly across institutions and publications. Reviewed training programs reported various measures of satisfaction, including completing a set number of robotic anastomoses on artificial or ex vivo models or meeting a set training duration on a specific robotic platform.

### 4.8 Limitations of Review

This review upholds several main limitations. Firstly, the underlying evidence base is limited in quality. The evidence is mostly low‐ to medium‐quality, with case series, mostly single‐surgeon experiences, and high heterogeneity across surgical techniques and platforms, limiting direct comparisons between some studies. The overwhelmingly small sample sizes also limit the evidence's statistical power. The possibility of publication bias within the field of robot‐assisted surgery should also be recognized, as challenges are often underreported. This is especially important given the high prevalence of authorships with conflicts of interest with robotic manufacturers MicroSure and MMI.

It is also important to recognize that these publications are geographically restricted to well‐resourced European and American institutions, and that they do not explore the challenges of introducing robotic platforms in poorly resourced areas. Accordingly, despite common themes across many articles, interpret and generalize conclusions with caution.

### 4.9 Future Directions

Future research could prioritize prospective comparative trials or, ideally, randomized controlled trials comparing manual microsurgery with a control group, including comparisons of patient outcomes and surgeon experience. These studies should evaluate not only short‐term complications but also long‐term patency and functional outcomes. This could provide higher‐quality evidence to guide risk–benefit discussions with patients.

Widespread reports of difficulties with limited haptic feedback and variable experience during the learning curve highlight the need to establish a standardized training pathway for future robotic microsurgeons. This extends to operating room staff, where dedicated training can target ergonomic inefficiencies and operating room set‐up.

Characterization of the learning curve across multiple surgeons remains limited, with most reports based on a single surgeon or a few highly experienced robotic surgeons. Future studies could standardize robotic platform training and consider prior microsurgical and robotic experience. It should aim to analyze the number of consecutive robot‐assisted microsurgical procedures required to reach a plateau in anastomotic times or other measures of technical performance. This will allow for a more accurate analysis of the learning curve independent of surgeon experience. Until such data become available, adopt robot‐assisted reconstructive microsurgery with awareness of the likely underestimated challenges identified in this review.

## 5 Conclusion

Robot‐assisted reconstructive microsurgery offers potential benefits, but implementation is constrained by prolonged operative times, increased costs, complex set‐up, absent haptic feedback, and variable training requirements. Evidence remains dominated by small, heterogeneous case series with limited long‐term outcomes. Prospective comparative studies, including randomized trials against manual microsurgery, should use standardized measures of operative efficiency, costs, learning curves, and long‐term patient outcomes to guide adoption.

## Author Contributions

**Lewei Cai:** methodology, investigation, formal analysis, writing – original draft. **Ishith Seth:** methodology, conceptualization, review and editing. **Sophie Ricketts:** conceptualization, review and editing. **Eldon Mah:** conceptualization, review and editing.

## Funding

The authors have nothing to report.

## Ethics Statement

The authors have nothing to report.

## Consent

The authors have nothing to report.

## Conflicts of Interest

The authors declare no conflicts of interest.

## Supporting information


**Table S1:** Search strategy applied for each database.
**Table S2:** Risk of bias assessment of case series performed through the JBI case series tool.
**Table S3:** Risk of bias assessment of randomized pilot studies performed through the RoB 2 tool.
**Table S4:** Risk of bias assessment of cross‐sectional studies performed through the JBI cross‐sectional studies tool.
**Table S5:** Risk of bias assessment of cohort studies and case–control studies performed through the Newcastle‐Ottawa scale.

## Data Availability

The authors confirm that the data supporting the findings of this study are available within the article or its Supporting Information.
