# Supplementary material for: Challenges of Robot‐Assisted Surgery in Reconstructive Microsurgery: A Scoping Review
Source: Microsurgery. 2026 Nov 10;46(8):e70339. doi: 10.1002/micr.70339 (PMC13749136; doi:10.1002/micr.70339)
Supplement: Supplementary file 1 [file MICR-46-e70339-s001.docx]

*Detailed Search Strategies*

| Data Base | Search String | Search Date Range | Number of Results |
| --- | --- | --- | --- |
| PubMed/Medline | (challenge*[tiab] OR limitation*[tiab] OR drawback*[tiab] OR learn*[tiab] OR barrier*[tiab] OR experience*[tiab] OR "Learning Curve"[Mesh])  AND  (robot*[tiab] OR Symani*[tiab] OR MUSA[tiab] OR MicroSure[tiab] OR "Robotics"[Mesh])  AND  (microsurg*[tiab] OR microvascular*[tiab] OR "free flap"[tiab] OR ((vascul*[tiab] OR lymph*[tiab]) AND anastom*[tiab]) OR nerve*[tiab] OR lymph*[tiab] OR "Microsurgery"[Mesh])  AND  (repair*[tiab] OR revascular*[tiab] OR reconstruct*[tiab] OR "Surgery, Plastic"[Mesh]) | 01/01/2016 to 30/09/2026 | 401 |
| Data Base | **Search String** | **Search Date Range** | **Number of Results** |
| Embase/Ovid | **1**  (challenge* or limitation* or drawback* or learn* or barrier* or experience*).ti,ab.  **2**  exp learning curve/  **3**  1 or 2  **4**  (robot* or Symani* or MUSA or MicroSure).ti,ab.  **5**  exp robotics/  **6**  4 or 5  **7**  (microsurg* or microvascular* or 'free flap' or ((vascul* or lymph*) and anastom*) or nerve* or lymph*).ti,ab.  **8**  exp microsurgery/  **9**  7 or 8  **10**  (repair* or revascular* or reconstruct*).ti,ab.  **11**  exp Surgery, Plastic/  **12**  10 or 11  **13**  (gastrointestin* or gastrectom* or colectom* or colorectal or colon* or rectal or rectum or bowel or intestinal or oesophag* or esophag* or gastric or stomach or hepato* or pancreatic or pancreat* or urolog* or prostate* or nephrectom* or kidney or renal or bladder or ureter* or urethra* or cystectom* or orchiectom* or testicular or penile or penis or scrot* or urinary).ti,ab.  **14**  (3 and 6 and 9 and 12) not 13  **15**  limit 14 to yr="2016 - 2026" |  | 294 |
| Data Base | **Search String** | **Search Date Range** | **Number of Results** |
| Cochrane | #1: ((challenge* OR limitation* OR drawback* OR learn* OR barrier* OR experience*)):ti,ab,kw  #2: MeSH descriptor: [Learning Curve] explode all trees  #3: #1 or #2  #4: ((robot* OR Symani* OR MUSA OR MicroSure)):ti,ab,kw  #5: MeSH descriptor: [Robotics] explode all trees  #6: #4 or #5  #7: ((microsurg* OR microvascular* OR "free flap" OR ((vascul* OR lymph*) AND anastom*) OR nerve* OR lymph*)):ti,ab,kw  #8: MeSH descriptor: [Microsurgery] explode all trees  #9: #7 or #8  #10: ((repair* OR revascular* OR reconstruct*)):ti,ab,kw  #11: MeSH descriptor: [Surgery, Plastic] explode all trees  #12: #10 or #11  #13: #3 and #6 and #9 and #12 | 01/01/2016 to 30/09/2026 | 43 |

*Supplementary Table S1. Search strategy applied for each database.*

Risk of Bias Assessments

| Study | Q1 | Q2 | Q3 | Q4 | Q5 | Q6 | Q7 | Q8 | Q9 | Q10 |
| --- | --- | --- | --- | --- | --- | --- | --- | --- | --- | --- |
| Lai et al., 2019^10^ |  |  |  |  |  |  |  |  |  |  |
| Barbon et al., 2022^4^ |  |  |  |  |  |  |  |  |  |  |
| Wittesaele & Vandevoort 2022^13^ |  |  |  |  |  |  |  |  |  |  |
| Lindenblatt et al., 2022^11^ |  |  |  |  |  |  |  |  |  |  |
| Daar et al., 2022^14^ |  |  |  |  |  |  |  |  |  |  |
| Beier et al., 2023^15^ |  |  |  |  |  |  |  |  |  |  |
| Weinzierl et al., 2023^17^ |  |  |  |  |  |  |  |  |  |  |
| Struebing et al., 2024^23^ |  |  |  |  |  |  |  |  |  |  |
| Wessel et al., 2024^20^ |  |  |  |  |  |  |  |  |  |  |
| Aman et al., 2024^24^ |  |  |  |  |  |  |  |  |  |  |
| Gorji et al., 2024^21^ |  |  |  |  |  |  |  |  |  |  |
| Struebing et al., 2024^6^ |  |  |  |  |  |  |  |  |  |  |
| Struebing et al., 2024^19^ |  |  |  |  |  |  |  |  |  |  |
| Kueckelhaus, 2024^18^ |  |  |  |  |  |  |  |  |  |  |
| Von Reibnitz et al., 2024^26^ |  |  |  |  |  |  |  |  |  |  |
| Chen et al., 2025^30^ |  |  |  |  |  |  |  |  |  |  |
| Piperno et al., 2025^29^ |  |  |  |  |  |  |  |  |  |  |
| Watson et al., 2025^28^ |  |  |  |  |  |  |  |  |  |  |
| Struebing et al., 2025^31^ |  |  |  |  |  |  |  |  |  |  |
| Struebing et al., 2025^27^ |  |  |  |  |  |  |  |  |  |  |
| Spille et al., 2025^33^ |  |  |  |  |  |  |  |  |  |  |
| Sørensen et al., 2025^34^ |  |  |  |  |  |  |  |  |  |  |
| Bettens et al., 2026^35^ |  |  |  |  |  |  |  |  |  |  |
| Struebing et al., 2026^36^ |  |  |  |  |  |  |  |  |  |  |

Q1. Were there clear criteria for inclusion in the case series?

Q2. Was the condition measured in a standard, reliable way for all participants included in the case series?

Q3. Were valid methods used for identification of the condition for all participants included in the case series?

Q4. Did the case series have consecutive inclusion of participants?

Q5. Did the case series have complete inclusion of participants?

Q6. Was there clear reporting of the demographics of the participants in the study?

Q7. Was there clear reporting of clinical information of the participants?

Q8. Were the outcomes or follow up results of cases clearly reported?

Q9. Was there clear reporting of the presenting site(s)/clinic(s) demographic information?

Q10. Was statistical analysis appropriate?

*Supplementary Table S2. Risk of bias assessment of case series performed through the JBI case series tool.*

| Study | D1 | D2 | D3 | D4 | D5 | Overall |
| --- | --- | --- | --- | --- | --- | --- |
| Van Mulken et al., 2020^5^ |  |  |  |  |  |  |
| Van Mulken et al., 2022^12^ |  |  |  |  |  |  |

D1. Randomisation.

D2. Deviations from intended interventions.

D3. Missing data.

D4. Outcome measurement.

D5. Selection of the reported result.

*Supplementary Table S3. Risk of bias assessment of randomised pilot studies performed through the RoB 2 tool.*

| Study | Q1 | Q2 | Q3 | Q4 | Q5 | Q6 | Q7 | Q8 |
| --- | --- | --- | --- | --- | --- | --- | --- | --- |
| Mayrhofer-Schmid et al., 2025^32^ |  |  |  |  |  |  |  |  |

Q1. Were the criteria for inclusion in the sample clearly defined?

Q2. Were the study subjects and the setting described in detail?

Q3. Was the exposure measured in a valid and reliable way?

Q4. Were objective, standard criteria used for measurement of the condition?

Q5. Were confounding factors identified?

Q6. Were strategies to deal with confounding factors stated?

Q7. Were the outcomes measured in a valid and reliable way?

Q8. Was appropriate statistical analysis used?

*Supplementary Table S4. Risk of bias assessment of cross-sectional studies performed through the JBI cross-sectional studies tool.*

| Study | D1 | D2 | D3 | Overall |
| --- | --- | --- | --- | --- |
| Shin et al., 2023^16^ |  |  |  |  |
| Dastagir et al., 2024^22^ |  |  |  |  |
| Moreira et al., 2024^25^ |  |  |  |  |

D1. Selection

D2. Comparability

D3. Outcome

*Supplementary Table S5. Risk of bias assessment of cohort studies and case-control studies performed through the Newcastle-Ottawa Scale.*
